# Transferrin Receptors in Erythropoiesis

**DOI:** 10.3390/ijms21249713

**Published:** 2020-12-19

**Authors:** Cyrielle Richard, Frédérique Verdier

**Affiliations:** 1 Inserm U1016, CNRS UMR8104, Institut Cochin, Université de Paris, 75014 Paris, France; cyrielle.richard@inserm.fr; 2 Laboratoire d’excellence GR-Ex, Université de Paris, 75014 Paris, France

**Keywords:** erythropoiesis, TFR1, TFR2, iron metabolism, iron uptake, transferrin

## Abstract

Erythropoiesis is a highly dynamic process giving rise to red blood cells from hematopoietic stem cells present in the bone marrow. Red blood cells transport oxygen to tissues thanks to the hemoglobin comprised of α- and β-globin chains and of iron-containing hemes. Erythropoiesis is the most iron-consuming process to support hemoglobin production. Iron delivery is mediated via transferrin internalization by the endocytosis of transferrin receptor type 1 (TFR1), one of the most abundant membrane proteins of erythroblasts. A second transferrin receptor—TFR2—associates with the erythropoietin receptor and has been implicated in the regulation of erythropoiesis. In erythroblasts, both transferrin receptors adopt peculiarities such as an erythroid-specific regulation of TFR1 and a trafficking pathway reliant on TFR2 for iron. This review reports both trafficking and signaling functions of these receptors and reassesses the debated role of TFR2 in erythropoiesis in the light of recent findings. Potential therapeutic uses targeting the transferrin-TFR1 axis or TFR2 in hematological disorders are also discussed.

## 1. Introduction

Erythropoiesis is a highly dynamic process wherein hematopoietic stem cells (HSCs) present in the bone marrow give rise to 2 × 10^11^ red blood cells (RBCs) per day [1]. This process involves several steps of differentiation, and is regulated by the niche and several cytokines and growth factors [2]. HSCs go through several differentiation steps to give rise to Burst Forming Unit–Erythroid cells (BFU-Es) which are entirely committed to the erythroid lineage. BFU-Es generate morphologically indistinguishable Colony Forming Unit–Erythroid cells (CFU-Es), whose proliferation capability is decreased. At this stage of differentiation, cell survival and proliferation become reliant on the main erythroid regulator erythropoietin (EPO) and on its receptor (EPOR) [2]. CFU-Es differentiate into proerythroblasts (Pro-E) which marks the beginning of terminal erythroid differentiation during which the synthesis of hemoglobin (Hb) takes place. Pro-E differentiate into early, then late basophilic erythroblasts (Baso-E1 then Baso-E2), which import iron for Hb production. Baso-E2s give rise to polychromatophilic erythroblasts (Poly-E) which already contain huge amounts of Hb. Poly-E divide into orthochromatic erythroblasts (Ortho-E) whose hemoglobinization is nearly completed. Ortho-E extrude their nuclei which is engulfed by the macrophage of the erythroblastic island in which terminal differentiation takes place [3], and give rise to reticulocytes which exit the bone marrow and mature into functional RBCs in the bloodstream. 

The ability of RBCs to transport oxygen is dependent on the Hb they contain. Hb is a heteroprotein complex containing α- and β-globin chains and iron-containing hemes to a 2:2:4 ratio in the adult. Due to the number of RBCs produced daily and the amount of Hb they contain (each cell carries around 1 × 10^8^ copies [4]), erythropoiesis is the most iron-consuming process [5]. 

Free iron may participate in oxidative reactions which generate toxic products [6]. However, in the serum, iron circulates as ferric ions complexed with the protein carrier transferrin (Tf), which allows the stable and safe transport of iron to the cells. Tf is bi-lobal and can bind one ferric ion per lobe [7]. Circulating Tf can be found devoid of iron (Apo-transferrin; Apo-Tf), with only one lobe occupied by iron or fully saturated (Holo-transferrin; Holo-Tf). Tf saturation reflects the body iron stores, and usually ranges between 20% and 45% in healthy individuals [7]. Holo-Tf has two receptors: transferrin receptor 1 (TFR1) and transferrin receptor type 2 (TFR2) [8], whose extracellular domains are highly homologous [9] and which are both expressed by erythroblasts [4]. Invalidation of either of these receptors leads to defective erythropoiesis [9,10,11], and polymorphisms in these genes are linked to red blood cell traits [12].

This review will focus on the transferrin receptors in erythroid cells, and their regulation dependent on iron sensing. An overview of these receptors as iron transporter and as mediator of cellular signaling pathway will be given, and their potential value as therapeutic targets in diseases affecting erythropoiesis and the iron metabolism will be highlighted.

## 2. Regulation of the Transferrin Receptors in Erythroid Cells

TFR1 and TFR2 are type II transmembrane glycoproteins, sharing 66% similarity in their extracellular domain. They form homodimers [8], thanks to cysteines forming disulfide bonds. Both are regulated by iron in erythroid cells as discussed below.

### 2.1. Regulation of TFR1

TFR1 (also called TFRC and cluster of differentiation 71 (CD71)) is encoded by the *TFRC* gene present on chromosome 3 in humans and 16 in *mus musculus* and is ubiquitously expressed. Despite its ubiquitous expression, the erythron is the tissue expressing the most TFR1 (2 × 10^6^ to 4 × 10^6^ copies per cell depending on the differentiation stage [4]). 

At the transcriptional level, TFR1 expression was found to be partly regulated by EPO thanks to the presence of STAT5 binding elements ((interferon-gamma–activated sequence (GAS) elements) in the first intron of *Tfrc* [13,14]. EPO is produced by the interstitial cells of the peritubular capillary bed of the kidneys when partial pressure of oxygen decreases [15]. By stimulating EPOR signaling, EPO ensures survival and proliferation of erythroblasts leading to the restoration of oxygenation through RBCs production. Interestingly, *TFRC* expression is also regulated by hypoxia, thanks to the presence of a hypoxia response element (HRE) in its promoter [16]. The hypoxia-inducible factor HIF-1α is stabilized in hypoxic condition or iron deficiency and binds to HREs leading to an increased expression of *TFRC* and *IRP1* (Iron Regulated Protein 1) among others. *TFR1* is also regulated post-transcriptionally in an intracellular iron-dependent manner through the Iron Responsive Element/Iron Regulated Protein (IRE/IRP) system [17]. When cellular iron is low, binding of Iron Regulated Proteins (IRP1 and IRP2) on the multiple IREs present within the 3′ UTR of TFR1 mRNA prevents its degradation, thus increasing its translation [18]. In case of high content of cellular iron, IRP1 activity switch to an aconitase activity with the binding of a Fe-S cluster while IRP2 is degraded [17]. TFR1 may also be post-translationally regulated by Apo-Tf which seem to decrease protein expression of TFR1 but not mRNA levels, however the mechanism of this regulation remains to be determined [19].

### 2.2. Regulation of TFR2

TFR2 is encoded by *TFR2* on chromosome 7 in humans and 5 in *mus musculus*. *TFR2* generates two isoforms—the canonical full length TFR2α, and a shorter isoform corresponding to the extracellular domain of TFR2α, called TFR2β [9,20]. Interestingly, an additional 142 bp can be found in the 5’ UTR of *TFR2-*β, possibly affecting its post-transcriptional regulation although no such regulation has been described yet. TFR2-β has been described as expressed ubiquitously [9] and due to the absence of transmembrane domain, it may be sequestered into the cytoplasm. It has been suggested to have a role in iron homeostasis in the spleen [21]. This review will focus solely on TFR2α, thereafter called TFR2. TFR2 expression is more restricted: it is highly expressed by the hepatocytes [9] wherein it participates in iron homeostasis [17], as well as in erythroblasts [4,10]. Recent reports also demonstrate its expression in macrophages [22], osteoblasts [23], and in neurons [24]. 

Several transcription factors have been described as promoting TFR2 expression, such as Gata1 which controls major erythroid genes and Cepb/α which is predominantly expressed in myeloid cells. The hepatocyte nuclear factor 4α has also been described to regulate *Tfr2* expression in hepatocytes [9]. Contrary to TFR1, TFR2 is not regulated by the IRE/IRP system. However, TFR2 expression is modulated post-translationally by its ligand, the Holo-Tf. RGD motif can be found on both TFR1 and TFR2 extracellular domain, mediating Holo-Tf binding [25]. In the case of TFR2, Holo-Tf binding leads to an increased expression in hepatic cells [26,27] and prevents cleavage of cell-surface TFR2 in erythroblasts [28]. The cell surface accumulation of TFR2 is the result of a shift from lysosomal degradation prompted by the tetraspanin CD81 to a recycling of the receptor to the plasma membrane [29,30,31]. As saturation of Tf reflects the level of body iron available, TFR2 cell-surface stability is regulated by systemic iron content.

### 2.3. Interaction with Their Ligand

While Holo-Tf can bind both Tf receptors, the affinity of TFR1 for Holo-Tf is 27 times greater than the affinity of TFR2 [8]. Both receptors present little to no affinity for Apo-Tf at pH 7.4. Partially saturated transferrin can be found in the circulation [32], ferric ions binding first to the C-terminal lobe of Tf [33]. Rabbit TFR1 can bind partially saturated transferrin although its affinity for C-lobe saturated transferrin and N-lobe saturated transferrin is only five times and six times greater than for Apo-Tf [34]. It is interesting to note that TFR2 affinity for partially saturated transferrin has not been characterized yet.

## 3. Iron Internalization and Transferrin Receptors

Iron is required for numerous biochemical processes such as metabolic and enzymatic activities, DNA repair, and oxygen transfer. In order to ensure this last point, 70% of the body iron can be found as hemoglobin in red blood cells [18]. 

### 3.1. Classical Internalization Pathway via TFR1

**Figure 1:**
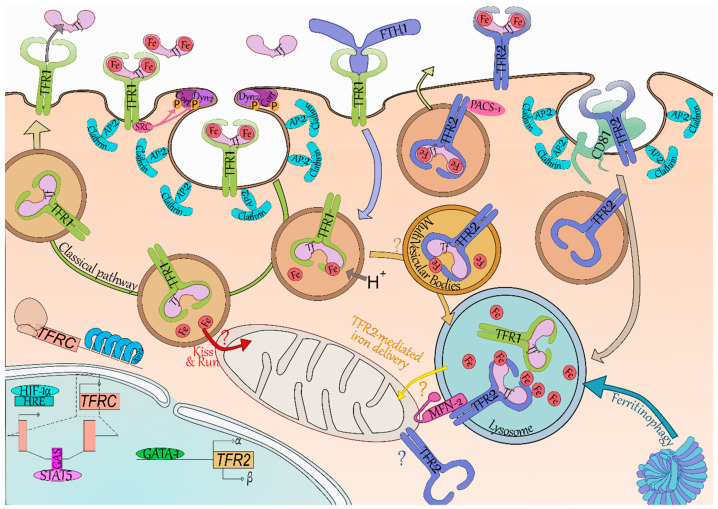
An erythroid-specific iron delivery pathway to mitochondria.

TFR1-mediated Tf internalization is classically described as the canonical iron import pathway (Figure 1). TFR1 is ubiquitously expressed but its expression is the highest in erythroblasts—in agreement with iron being needed ubiquitously but mainly consumed by erythropoiesis. This is illustrated by the phenotype of *Tfr1^−/−^* mice which die in utero due to severe anemia leading to a defect in RBCs productions [11].

Holo-Tf binds to TFR1 which contains a ^20^YTRF^23^ internalization motif. The Holo-Tf-TFR1 complex is then internalized by clathrin-mediated endocytosis [35,36] through the recruitment of Dyn2 and Cort phosphorylated by Src kinase [37]. TFR1 clustering promotes coated pit initiation [38]. Endosome acidification then leads to the dissociation of iron from Tf, but Apo-Tf remains bound to TFR1 [17]. The Tf-TFR1 complex is found in the early endosomes, and is mainly recycled [35]. At physiological pH found at cell-surface, Tf is released from TFR1 into the serum where it may bind ferric ions once more. Internalization of Tf and recycling of TFR1-Tf complex was shown to take 16 min on average in a human hepatoma cell line [39]. 

In erythroid cells, iron is mainly used for hemoglobin synthesis. Due to iron requirement of these cells, they contain a substantial amount of iron which would trigger the IRE/IRP system in other cell types. Indeed, in iron replete condition, a functional IRE/IRP system leads to TFR1 decreased expression. This system was found nonfunctional in differentiating erythroid cells [40], allowing high and continuous expression of TFR1 all along erythroid differentiation [4]. It has been suggested that endosomes containing Holo-Tf-TFR1 come close to mitochondria and deliver iron directly to this organelle via a “kiss-and-run” mechanism (Figure 1)—thus limiting the cytoplasmic iron pool and not triggering the regulation by the IRE/IRP system [41].

The iron carrier transferrin (Tf, light pink) can bear one ferric ion (Fe, red) on each of its two lobes, and can be found in the circulation with either lobe occupied, both or none. TFR1 (yellow green) and TFR2 (blue) have a greater affinity for bi-loaded Tf and little to none for unloaded Tf at pH 7.4. The **classical pathway** (green arrow) of iron internalization is through binding to TFR1. SRC kinase (dark pink) is bound to TFR1 and recruits cortactin (Cort, dark purple) and dynamin 2 (Dyn2, light purple) through phosphorylation (P, orange). Coated pit initiation and AP-2 mediated recruitment of clathrin (light blue) leads to internalization of the Iron-Tf-TFR1 complex. Acidification (H^+^) of the endosome (brown vesicle) leads to iron release from Tf. In erythroid cells, it has been suggested the ferric ions directly enter the mitochondria—thus limiting iron-related oxidative stress in the cytoplasm (“**Kiss and Run**”, red arrow and “?”). At acidic pH, TFR1 readily binds Tf preventing the dissolution of their connection. The Tf-TFR1 complex is recycled back to the membrane where the neutral pH leads to Tf disengagement from TFR1. The heavy chain of ferritin (FTH1, periwinkle blue) has been found to also bind and be internalized by TFR1. FTH1 internalized through TFR1 was found in the lysosome (blue vesicle), which is also responsible for iron release from conventional ferritin cages (dark and light blue complex) through the process called **Ferritinophagy**. TFR2 degradation (light grey arrow) occurs in the lysosome and seems reliant on the tetraspanin CD81 (tea green). Binding of iron-loaded Tf to TFR2 increases TFR2 half-life and induces a shift from degradation to a recycling pathway. Recycling of TFR2 seems promoted by the phosphofurin acidic cluster sorting protein 1 (PACS-1, dark pink). In erythroid cells, TFR2-bound transferrin was localized in the multivesicular bodies (mellow yellow vesicle) then in the lysosome. As FTH1 internalized through TFR1 also ends up in this compartment, we do not exclude the possibility of a transfer from TFR1 internalization-mediated endosomes to TFR2-containing multivesicular bodies/lysosomes (light orange arrow and “?”). The mitochondrial outer membrane protein Mitofusin-2 (MFN-2, magenta) is involved in mitochondria-lysosome contacts and associates with TFR2. Through this association, TFR2 could route iron in the mitochondria although the exact mechanism remains to be clarified (“**TFR2-mediated iron delivery**”, yellow arrow and “?”). Of note, TFR2 has been found to be mitochondrial in neurons and therefore may also be in erythroid cells (blue “?”). In terms of gene expression, *TFR2* (khaki box) expression is driven through the erythroid transcription factor GATA binding protein 1 (GATA-1, grass green) and generates the α and β isoforms—although no protein evidence has been found as of yet for the latter. *TFRC* (encoding TFR1, light red) is regulated by hypoxic level through the hypoxia-inducible factor 1-α (HIF-1α, electric blue) binding to hypoxia response element (HRE, powder blue), as well as by the binding of Signal transducer and activator of transcription 5 (STAT5, pink) on interferon-gamma–activated sequence elements (GAS, deep pink). *TFRC* is also regulated at the post-transcriptional level by the Iron Responsive Element/Iron Regulatory Protein (IRP1/2, blue) system which stabilizes its mRNA in iron-depleted condition.

### 3.2. TFR1 Internalizes Iron-Loaded Ferritin

Li et al. demonstrated TFR1 can also import iron bound to the heavy chain of ferritin (FTH1) (Figure 1) [42]. This was later confirmed by Sakamoto et al. which suggested this is especially the case in erythroid cells [43]. Ferritins enable intracellular iron storage by forming ferritin cages consisting of 24 subunits of heavy (FTH1) and light (FTL) chains in varying ratios depending on cell types [44]. Ferritin cages contain up to 4500 ferric ions. Both FTH1 and FTL expression is repressed in case of iron deficiency by the IRE/IRP system thanks to IRE in their 5’ UTR [17]. Ferritins may be released in the serum, in particular by macrophages which participate in systemic iron storage and recycling. Ferritins secreted by the macrophages of the erythroblastic islands could supply nearby erythroblasts in iron via TFR1 [42,45]. FTH1 binding site on TFR1 is distinct from the binding site of Holo-Tf, and TFR1-mediated FTH1 uptake requires a greater expression of TFR1 to be internalized. This suggests that more than one TFR1 complex is necessary and could explain why only erythroblasts efficiently take up FTH1 [42,43]. Internalization of the FTH1-TFR1 complex has been less studied than Holo-Tf-TFR1, however, there is evidence that FTH1 enters both endosomes and lysosome upon internalization [42]. The ability of TFR1 to bind and internalize both iron-loaded carriers ensure suitable iron supply to erythroblasts.

### 3.3. An Erythroid Specific Iron Delivery Pathway to Mitochondria 

Tfr2 was first thought uninvolved in iron import in erythroblasts as it does not make up for Tfr1 absence in *Tfrc^–/–^* mice [11], however it may be involved in intracellular iron trafficking as recently described in erythroid cells [46]. Khalil et al. demonstrated that most of the transferrin is found in the MVB/lysosome compartment in these cells rather than in the recycling endosomes [46]. TFR2 has a mitochondrial targeting sequence and associates with MFN2 which is involved in mitochondrial-lysosomal contacts (Figure 1). By mediating direct contacts between these two organelles, TFR2 could contribute to erythroid iron delivery to the mitochondria. This novel pathway may allow iron transfer into the cell without iron-mediated oxidative stress in the cytoplasm. Interestingly, a similar mitochondrial iron transport pathway has been described in neurons [47]—which are also extremely reliant upon iron uptake as illustrated by the neural defects present in *Tfrc^–/–^* mice [11]. 

Cell surface TFR2 internalization rate is extremely low compared to TFR1 [31,39], which in addition to its weaker affinity for Holo-Tf compared to TFR1’s [8], and its weaker expression by erythroblasts [4] suggest TFR2 is not responsible for most of the iron import. However, with this recent data, it is possible to consider another model using both transferrin receptors to import iron in erythroid cells. TFR1-Tf is known to be found in endosomes after coated-pit mediated internalization [35], and these could be routed directly to the mitochondria via a “kiss-and-run” mechanism [41]. Additionally, TFR1-Tf containing endosomes may be targeted to the MVBs, wherein they may join the TFR2-dependent routing pathway directed to the lysosome. TFR2 may then participate in the shuttling of the iron internalized through TFR1 (retrieved from both Holo-Tf and FTH1), ensuring it is delivered directly to the mitochondria by establishing lysosomal-mitochondrial contacts (Figure 1).

## 4. Signaling of the Transferrin Receptors and Erythropoiesis Modulation

While transferrin receptors are involved in iron import or iron routing into the cell, several studies suggest their role as signal mediators.

### 4.1. TFR1 Signaling

**Figure 2:**
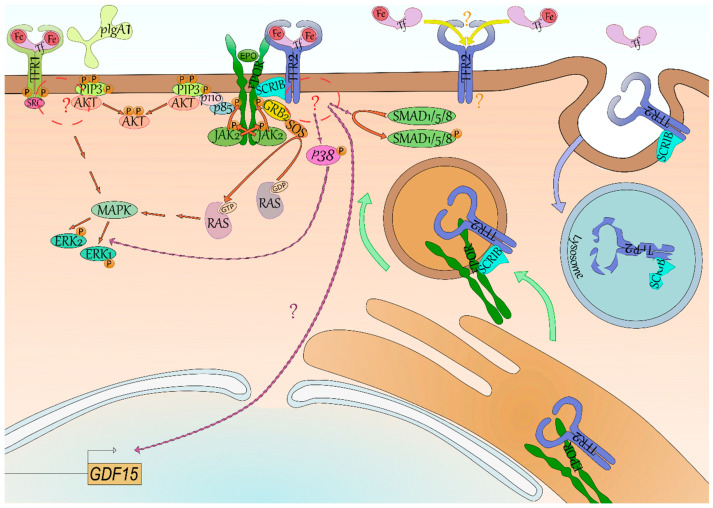
Signaling of the transferrin receptors.

Evidence for signaling properties of TFR1 is scarce. In breast cancer cells, the apoptosis-inducing agent gambogic acid induces the activation of Src kinase that may phosphorylate the Y^20^ of the receptor leading to the activation of signaling pathways implicating MAPK/ERK, Caspase 3, and Caspase 8 [48] (Figure 2). While the use of gambogic acid is not physiological, phosphorylation of Y^20^ has been found to induce signaling downstream of TFR1 in response to Holo-Tf and to polymeric IgA1 in erythroid cells [49]. Polymeric IgA1 (pIgA1) is produced mainly in the bone marrow and is enhanced in hypoxic condition. The interaction of pIgA1 with TFR1 supports the maintenance of human erythropoiesis by inducing the MAPK/ERK and PI3K/AKT pathways synergically with EPO (Figure 2). Binding of Holo-Tf to TFR1 induces these same signaling pathways, however, pIgA1 does not compete with Holo-Tf for TFR1 binding [49].

Iron-loaded transferrin (Tf, light pink) and polymeric IgA1 (light green) have been found to induce phosphorylation (P, orange) of the Y^20^ of TFR1 (yellow green) by the Src kinase (dark pink) through their binding. Both phosphatidylinositol trisphosphate (PIP3, lime green)/AKT (light red) and Mitogen-Activated Protein Kinase (MAPK, mantis green)/Extracellular signal-Regulated Kinase (ERK, mint green) signaling seem activated downstream of TFR1, although the intermediates between TFR1-SRC and AKT/MAPK remain unknown (red circle and “?”). Signaling downstream of TFR2 (blue) is even more elusive—GDF15 (light brown box) expression is reliant on TFR2 in erythroid cells but the signaling is undefined (lilac arrow and “?”). Some studies suggest the activation of p38 (pink)/ERK and of SMAD1/5/8 (green). Signaling of its erythroid partner, the erythropoietin receptor (EPOR, dark green), is better characterized with the binding of erythropoietin (EPO, grass green) leading to trans-phosphorylation of its associated kinase Janus Kinase 2 (JAK2, light green) and to phosphorylation of EPOR. Mediator proteins such as Growth factor Receptor-Bound protein 2 (GRB2, yellow) and p85 (periwinkle blue) bind to these docking sites and through Son Of Sevenless (SOS, orange) and p110 (light pink), respectively, lead to the activation of RAS (inactive grey, active silver pink)/ERK and PI3K/AKT signaling. Recently, the SCRIBBLE protein (SCRIB, ice blue) has been shown to associate with both EPOR and TFR2 in erythroid cells and to promote EPOR export to cell surface (green arrow). In case of low saturation of Tf, TFR2 is not stabilized at cell surface and both TFR2 and SCRIB are degraded (blue arrow) by the lysosome (blue vesicle)—SCRIB is then not available anymore for EPOR export to cell surface. Lastly, it has been suggested in mice that differential occupancy of Tf lobes induces a distinct effect on erythropoiesis and EPO sensibility. One hypothesis explaining these results is that TFR2 has contrasting affinity for N-ter and C-ter Tf lobe occupancy—it however remains to be characterized (yellow arrow and “?”). 

### 4.2. TFR2 Signaling

Contrary to TFR1, the signaling of TFR2 remains mostly elusive. In erythroid cells, TFR2 low expression compared with TFR1 and its weaker affinity for Holo-Tf makes it an ideal candidate for transferrin saturation sensing. Accordingly, binding of Holo-Tf to TFR2 in the hepatocyte leads to the production of the iron lowering hormone hepcidin implying the existence of a signaling downstream of TFR2 [9]. Aside from *HAMP* (encoding hepcidin) in the hepatocyte [9], *GDF15* production was directly dependent on a TFR2-mediated signaling in erythroblasts as well [10] (Figure 2). 

While several studies have been conducted, signaling downstream of Tfr2 in erythroid cells remains poorly described. Calzolari et al. observed the signaling p38/ERK was stimulated with an antibody mimicking binding of Holo-Tf to TFR2 in the erythroleukemic cell line K-562 [50]. While this first study used a nonphysiological ligand, it was supported by other studies in other cell types. Rauner et al. recently described the activation of the p38/ERK axis downstream of Tfr2 in osteoblasts as well. They also observed a decreased phosphorylation of Smad1/5/8 in their knockout model [23]. Interestingly, a cross-talk has previously been suggested between the Mapk/Erk and Smad1/5/8 pathways in hepatic cells [51]. TFR2-mediated activation of Smad1/5/8 was never reported in erythroid cells and remains cryptic in nonerythroid cells. Indeed, in hepatocytes, both SMAD1/5/8 and p38/ERK were found affected in absence of Tfr2 [51,52,53], however it was not the case in every study [54]. 

### 4.3. Erythropoiesis Modulation through the TFR2-EPOR Complex

Aside from its own signaling, TFR2 may also affect EPOR signaling in erythroid cells by regulating EPOR cell surface expression [32,55]. Forejtnikovà et al. demonstrated that TFR2 and EPOR associate in the endoplasmic reticulum of erythroblasts and that TFR2 then facilitates the transport of EPOR to cell surface. Accordingly, *Tfr2^−/−^* CFU-Es were found less sensitive to Epo potentially due to a decreased of Epor at their surface [10]. Recently, Khalil et al. described the scaffold protein SCRIBBLE as an escort for EPOR whose presence is dependent on transferrin saturation via its association with TFR2 [55]. In low saturation condition, there is no Holo-Tf available to bind TFR2 which is internalized and degraded in the lysosome [31]. SCRIBBLE is degraded alongside TFR2 by the lysosome (Figure 2), and is not available anymore for EPOR transport at cell surface leading to iron-restricted erythropoiesis [55]. Therefore, aside from a role as an iron importer to mitochondria and its own signaling, TFR2 is also important for EPOR export to cell surface in an extracellular iron-dependent manner. 

### 4.4. TFR2 and Erythropoiesis Modulation

Deletion of *Tfr2* in mice leads to an acute iron overload called hemochromatosis [9] mimicking the human disease hereditary hemochromatosis type 3 due to *TFR2* mutations [56]. This pathology is solely due to the absence of hepatic TFR2. The first studies did not focus on erythroid cells since neither murine models nor human patients affected with hemochromatosis type 3 show abnormal erythroid parameters. In 2010, the role of TFR2 in erythropoiesis was reported for the first time. A delayed differentiation of human primary erythroblasts knocked down for *TFR2* expression and a decreased sensibility to Epo of murine erythroid progenitors CFU-E from *Tfr2^−/−^* mice were observed [10]. Following this study, contradictory observations were made in mouse models, with the knockout of *Tfr2* leading either to a greater production of RBCs [57,58], or to a decreased production [59,60]. Recently, several studies explored the effect of erythroid *Tfr2* deletion in bone-marrow specific deletion models with once again varying results. Indeed, an enhanced erythropoiesis was observed by Nai et al. [61] while a delayed erythroid differentiation was reported by Rishi et al. [62]. The differences observed can partially be explained by the authors not using mice of the same gender nor age, as well as of various mouse strains [63]. One of the main differences in the set up of these studies, however, is the amount of iron found in the mice diet, and whether a restricted diet was enforced since birth or after several months [61,62]. As Tfr2 acts as an iron sensor, its signaling is likely dependent on transferrin saturation which reflects the body iron status. As such, it is plausible that differing iron status would modulate the phenotype observed in absence of Tfr2. The manner in which the bone-marrow specific deletion of *Tfr2* was obtained in these studies should also be considered. The bone marrow transplantation used by Nai et al. leads to deletion of *Tfr2* in the niche as well as in erythroblasts [61] while the use of *Vav1* restricted Cre recombinase as used by Rishi et al. does not affect as many cell types [62,64]. Macrophages have been described to express Tfr2 [22], and deletion of Tfr2 in the macrophages of the erythroblastic island may affect erythropoiesis. 

Only one study exploring the role of erythroid TFR2 in human was performed, using ex vivo culture of primary erythroblasts [10]. Considering the higher expression of TFR2 in human erythroblasts compared with murine erythroblasts [4,65], further studies in human models such as the human erythroid cell lines BelA [66] or Hudep2 [67] would help to better understand the role of TFR2 in erythropoiesis. Additionally, in mice models, erythropoiesis may be highly compensated by stress erythropoiesis which generates a high number of erythrocytes to maintain homeostasis. This process of stress erythropoiesis is particularly efficient in mice, in which it takes place mainly in the spleen, as well as in the bone marrow, and the liver [68]. Ex vivo or in vitro systems may offer additional information which may be partially occluded in systemic models, thereby helping to define the role of TFR2 in erythropoiesis.

### 4.5. Differential Lobe Occupancy of Transferrin as a Signaling Cue 

Recently, Parrow et al. have described differing role of lobe C-loaded transferrin (C-Tf) and N-loaded transferrin (N-Tf) [32]. They established mouse models with transferrin only able to bind one lobe out of two. Both C lobe-restricted and N lobe-restricted mice have iron deposits in the liver and a decreased hepcidin expression relative to iron content although this is more pronounced in the C lobe-restricted model. Both models also present some aspects of iron-restricted erythropoiesis such as decreased hemoglobin levels and microcytosis. However, C lobe-restricted mice have a more drastic drop in hemoglobin level than the N lobe-restricted model, which have a greater number of RBCs. Interestingly, C lobe-restricted mice had an increase in serum Epo compared with N lobe-restricted or WT mice, and a decrease phosphorylation of AKT in erythroblasts. Treatment of these mice with Epo demonstrated a higher Epo responsiveness for the N lobe-restricted model (increased number of RBCs, increased level of hemoglobin), demonstrating that iron occupancy of the N lobe of transferrin is implicated in Epo responsiveness. This could be explained by N lobe occupancy reflecting iron sufficiency as it is loaded after the C-lobe as explained previously [33]. While TFR1 affinity for partially loaded transferrin has been described previously, TFR2 affinity for C-Tf or N-Tf has not been investigated yet. The differing responsiveness to Epo in these models could be due to TFR2 association with EPOR. A higher affinity of TFR2 for N-Tf compared to C-Tf could lead to a higher recycling of TFR2 [31], which could lead to an increased expression of EPOR at cell surface and a higher responsiveness [10,55]. 

## 5. Transferrin Receptors as Clinical Markers and Therapeutic Targets

As both transferrin receptors are regulated in some way by iron levels (*cf* 2.1, 2.2), and various pathologies affect both iron metabolism and erythropoiesis, their expression is relevant for diagnostic and directed therapy.

### 5.1. Shedding of the Transferrin Receptors—A Clinical Marker

Both transferrin receptors possess a protease associated domain which upon cleavage leads to the release of their extracellular domain in the serum. Upon recycling, TFR1 is hydrolyzed between residues Arg100 and Leu101 leading to the release of its extracellular domain from the cell into the blood as a soluble TFR1 (sTFR1). The cleaved intracellular domain of TFR1 may also be released in the serum [69]. The concentration of sTFR1 reflects erythropoiesis rate and iron depletion [70]. Indeed, circulating sTFR1 level reflects total body TFR1 concentration. Expansion of erythroid precursors to compensate a loss of RBCs lead to an increased level of sTFR1 due to an increase of highly expressing TFR1^+^ cells. As a consequence, iron deficiency anemia is associated with high concentrations of sTFR1, as in several pathologies such as β-thalassemia or hemolytic anemia [70]. sTFR1 levels are used as a predictive marker for diagnosis [71].

While sTFR1 was described as early as in 1983, the soluble form of TFR2 was described more recently. In 2015, Pagani et al. demonstrated in vitro a soluble form of TFR2 (sTFR2) is released from erythroid cells. Holo-Tf inhibits TFR2 cleavage in a dose-dependent manner. Therefore, iron-deficient conditions lead to an increased release of sTFR2. As sTFR1, it is a marker of iron deficiency. In immortalized hepatic cell line, sTFR2 seems to inhibit the activation of *HAMP* promoter. However, it does not seem to affect EPOR signaling [28]. Further studies are required to determine the functional role of sTFR2 in vivo.

### 5.2. Expression of the Transferrin Receptors as Prognostic Marker 

Many types of blood cancer have a very poor prognosis, and the use of markers to predict the patient’s survival is crucial to adapt therapeutic strategies to the individual. The level of expression of TFR2 is a good indicator for patient survival in a few types of blood cancer. Myelodysplastic syndromes (MDS) are a group of heterogeneous malignant hematologic disorders characterized by ineffective hematopoiesis and peripheral blood cytopenia. These syndromes are pre-leukemic and are associated with poor clinical outcome. In some cases, MDS may progress into acute myeloid leukemia (AML), which is more aggressive [72]. In 2017, Di Savino et al. showed that patients presenting a high grade MDS and a reduced expression of TFR2 had a poorer survival [73]. Accordingly, AML patients presenting a high level of TFR2 expression at the mRNA level lived longer [74]. TFR1 expression was not linked to survival in AML patients [74,75], but a high expression of TFR1 was associated with thrombocytopenia, severe anemia, and the prevalence of complex aberrant cytogenetics [75].

### 5.3. TFR1 as a Therapeutic Target 

As cell proliferation requires iron for DNA replication, TFR1 expression is generally increased in cancer cells compared to their normal counterparts [76]. In a nonpathological context, erythroblasts are the most TFR1 expressing cells. Several antibodies directed against TFR1 able to hinder its activity were developed. Antibody binding was shown to reduce proliferation and to induce cytotoxicity in T-cell leukemia [77]. Several strategies exploiting the role of TFR1 as an importer were also attempted to specifically target cancer cells and erythroblasts. For example, anti-TFR1 antibodies complexed with liposomal nanoparticles containing the wild-type version of the human oncogene p53 were shown to successfully deliver their cargo into pancreatic tumors in mice and led to an increase in survival [78]. TFR1 was also used as a “Trojan horse” to internalize antisense oligonucleotide into erythroid cells ex vivo—leading to the restoration of the previously deficient Ferrochelatase activity in a patient with erythropoietic protoporphyria [79].

As TFR1 is a receptor for FTH1 as well [42], and TFR1-mediated internalization of FTH1 is dependent upon a high expression of TFR1 [43], drug molecules could be encapsulated in ferritin cages for delivery directly to cancer cells [80] in a potentially more specific manner than with the previous methods. However, while cancer therapies targeting TFR1 or using TFR1 as a gate for drug delivery are promising, their application as therapeutic means will require careful consideration to avoid a toxic targeting of the noncarcinogenic erythroid cells expressing highly TFR1. 

Lastly, TFR1 has been described previously as an entry receptor for arenaviruses and for *Plasmodium vivax*, a parasite invading preferentially reticulocytes [81,82]. Recently, Gruszczyk et al. reported that the interaction between TFR1 and PvRBP2b is crucial for the initial recognition of reticulocytes by *P. vivax*, offering potential new therapeutic avenues to inhibit the blood stage propagation of the parasite [81].

### 5.4. Transferrin, Transferrin Receptors, and β-Thalassemia

β-thalassemias are caused by mutations in the *HBB* gene encoding β-globin. Due to a decreased production of β-globin chains, α-globin chains are in excess and form aggregates causing oxidative stress and apoptosis of erythroblasts leading to ineffective erythropoiesis. Together with a shortened lifespan of RBCs, patients present a mild to severe anemia [83]. Consecutive to anemia, hypoxia induces EPO synthesis leading to stress erythropoiesis in bone marrow and spleen in an attempt to compensate the erythroid disorder. The erythron increased iron requirement leads to hepcidin suppression and parenchymal iron deposits. Patients requiring transfusions to maintain tissue oxygenation also face transfusional iron loading which worsen the symptoms. Transferrin saturation is greatly increased, and nontransferrin bound iron accumulates causing tissues damage. Iron overload accounts for most of the comorbidities observed in this disease, thus strategies inducing iron-restriction and stimulating erythropoiesis could lessen the symptoms [83]. 

Li et al. showed in 2010 that treating β-thalassemia intermedia mice (*Hbb^th3/+^*) with transferrin mimics iron-restricted erythropoiesis which is characterized with low mean corpuscular volume and low hemoglobin due to decreased iron delivery to erythroid precursors [84]. Transferrin-treated β-thalassemic mice presented an improved erythropoiesis due to the decreased iron import. As globin production is transcriptionally regulated by heme availability, depending itself on iron content [85], transferrin treatment results in fewer α-globin aggregates and thus in a decreased apoptosis of erythroblasts and a lengthened lifespan of RBCs. As a consequence, these mice had more circulating RBCs, and systemic iron overload was decreased.

With its central role in iron metabolism and erythropoiesis regulation, TFR2 has also been proposed as a therapy target for this disease [86]. In 2018, Artuso et al. found that the bone-marrow specific deletion of *Tfr2* moderately lessens the symptoms of β-thalassemia in their *Hbb^th3/+^* mouse model, when combined with an iron restricted diet [87]. More recently, a study from Casu et al. combined *Tfr2* knockout with iron depletion by targeting the negative regulator of Hepcidin (*Tmprss6*) which shows superior correction of anemia in *Hbb^th3/+^* mice [88]. In both studies, the number of RBCs and the hemoglobin level are increased and iron deposits in the liver, spleen, and kidney are decreased. While the authors postulate this effect is due to an increased sensibility to Epo, iron restriction due to decreased Tfr2 is just as likely and is further increased with the targeting of *Tmprss6*. Indeed, it was demonstrated previously that *TFR2* knockdown leads to a decreased EPO sensibility [10] and TFR2 may be required for iron import to mitochondria in erythroblasts [46]. As iron deprivation seems to ameliorate the symptoms, this is an alternate explanation to consider. These studies in the context of β-thalassemia intermedia have been done with the same mouse models [87,88]. However, varying results have been obtained with *Tfr2* knockout depending on the mouse model before [10,59,61,62], probably partly due to differing basal level of iron [63]. Additionally, these studies focused on a model of β-thalassemia intermedia which is not dependent on transfusion. Assessing the effect of *Tfr2* knockout in β-thalassemia major and furthering the understanding of the mechanism by which *Tfr2* deletion affects β-thalassemia are required prior to the implementation of a clinical application.

## 6. Conclusions

The transferrin receptors play pivotal roles in erythropoiesis by sustaining both iron delivery and signaling. Regulation of these receptors by intracellular (TFR1) and systemic (TFR2) iron levels make them important actors in the interaction between iron metabolism and erythropoiesis. Along with their ligand transferrin, they are interesting potential molecular targets for diagnosis and treatment of diseases. Due to their carrier action, transferrin receptors may be targeted for their function as well as for their ability to import treatments into the cell. Their expression pattern makes them particularly relevant for treatment of diseases affecting both iron metabolism and erythropoiesis such as β-thalassemia. However further studies are required to understand the role of TFR2 in normal and iron restricted erythropoiesis and the clinical implication of its targeting in β-thalassemia in conjunction with other strategies such as Apo-Tf treatment.

## Abbreviations

AML	Acute myeloid leukemia
Apo-Tf	Apo-transferrin
Baso-E1	Early basophilic erythroblast
Baso-E2	Late basophilic erythroblast
BFU-E	Burst Forming Unit–Erythroid cells
CFU-E	Colony Forming Unit–Erythroid cells
C-Tf	C-lobe loaded transferrin
EPO	Erythropoietin
EPOR	Erythropoietin receptor
FTH1	Ferritin heavy chain
FTL	Ferritin light chain
Hb	Hemoglobin
Holo-Tf	Holo-transferrin
HRE	Hypoxia response element
HSCs	Hematopoietic stem cells
IRE/IRP	Iron Responsive Element/Iron Regulated Protein
MDS	Myelodysplastic syndrome
MVB	Multivesicular bodies
N-Tf	N-lobe loaded transferrin
Ortho-E	Orthochromatic erythroblast
pIgA1	Polymeric immunoglobulin A1
Poly-E	Polychromatophilic erythroblast
Pro-E	Proerythroblast
RBCs	Red blood cells
RGD	Asparagine-Glycine-Aspartic acid
sTFR1	Soluble TFR1
sTFR2	Soluble TFR2
Tf	Transferrin
TFR1	Transferrin receptor 1
TFR2	Transferrin receptor type 2
UTR	Untranslated region
YTRF	Tyrosine-Threonine-Arginine-Phenylalanine
